# Chemopreventive Agents and Inhibitors of Cancer Hallmarks: May *Citrus* Offer New Perspectives?

**DOI:** 10.3390/nu8110698

**Published:** 2016-11-04

**Authors:** Santa Cirmi, Nadia Ferlazzo, Giovanni E. Lombardo, Alessandro Maugeri, Gioacchino Calapai, Sebastiano Gangemi, Michele Navarra

**Affiliations:** 1 Department of Chemical, Biological, Pharmaceutical and Environmental Sciences, University of Messina, Messina I-98168, Italy; scirmi@unime.it (S.C.); nadiaferlazzo@email.it (N.F.); maugeri.alessandro@gmail.com (A.M.); 2 Department of Health Sciences, University “Magna Graecia” of Catanzaro, Catanzaro I-88100, Italy; gelombardo@unicz.it; 3 Department of Biomedical and Dental Sciences and Morphofunctional Imaging, University of Messina, Messina I-98125, Italy; gcalapai@unime.it; 4 Department of Clinical and Experimental Medicine, University of Messina, Messina I-98125, Italy; gangemis@unime.it; 5 Institute of Applied Sciences and Intelligent Systems (ISASI), National Research Council (CNR), Pozzuoli I-80078, Italy

**Keywords:** *Citrus*, cancer, flavonoids, nutraceuticals, functional foods, natural product, complementary and alternative medicines

## Abstract

Fruits and vegetables have long been recognized as potentially important in the prevention of cancer risk. Thus, scientific interest in nutrition and cancer has grown over time, as shown by increasing number of experimental studies about the relationship between diet and cancer development. This review attempts to provide an insight into the anti-cancer effects of *Citrus* fruits, with a focus on their bioactive compounds, elucidating the main cellular and molecular mechanisms through which they may protect against cancer. Scientific literature was selected for this review with the aim of collecting the relevant experimental evidence for the anti-cancer effects of *Citrus* fruits and their flavonoids. The findings discussed in this review strongly support their potential as anti-cancer agents, and may represent a scientific basis to develop nutraceuticals, food supplements, or complementary and alternative drugs in a context of a multi-target pharmacological strategy in the oncology.

## 1. Introduction

Cancer and heart disease are two of the main pathologies worldwide, and the most common causes of death in old age. The decline in death rates over the last century has resulted in a large proportion of people beginning to live up to eighty years old or more, and an increased incidence of chronic diseases. Thus, cancer represents a crisis for public health, with an estimated 14 million cases globally with a total of 8.2 million deaths for cancer in 2012 [1]. The two most important ways to reduce cancer risk are the avoidance of cancer-causing agents and finding preventive strategies to stop cancer onset. Obviously, death to cancer can be reduced by the discovery of new drugs or novel therapeutic approaches, designed to stop the development of clinical cancer in the first instance.

Despite the ongoing development of synthetic drugs that represent the mainstay of pharmaceutical care, the plant kingdom still remains an attractive source of novel anti-cancer drugs. It provides biologically active molecules for use in pharmaceuticals applications, and it has been estimated that about 70% of anti-cancer drugs originate to some extent from natural sources [2]. Moreover, both observational and experimental studies suggest that regular consumption of fruits and vegetables may play an important role in reducing degenerative diseases such as cancer [3,4,5]. Recently, it has been suggested that, among tissues, a third of the variation in cancer risk is attributable to environmental factors or hereditary predisposition, and that changes in lifestyle can play a very important role in the development of certain types of cancer [6]. About 30%–40% of cancer incidence could be prevented by an healthy diet, doing regular physical activity, and maintaining correct body weight [7]. Overall, a high dietary intake vegetables and fruits (>400 g/day) could prevent at least 20% of all cancer cases [7,8].

The cancer protective effects of vegetables and fruits may be due to the presence of bioactive molecules acting through different mechanisms including the following: inhibition of carcinogen activation, stimulation of carcinogen detoxification, scavenging of free radical species, control of cell-cycle progression, inhibition of cell proliferation, induction of apoptosis, inhibition of oncogene activity of, inhibition of angiogenesis and metastasis, and inhibition of hormone or growth-factor activity [4,9,10,11,12].

*Citrus* fruits (CF), i.e., oranges, lemons, limes, bergamot, grapefruits, and tangerines, are popular all over the world. CF are the main winter fruits consumed in the Mediterranean diet, meaning they are the main source of dietary flavonoids. They are rich in vitamins and flavonoids, and have long been hypothesized to possess a protective effect against cancer.

This review is an attempt to provide an insight into the anti-cancer effects of CF, with a focus on their bioactive compounds, elucidating the main cellular and molecular mechanisms by which they may protect against cancer.

## 2. The *Citrus* Flavonoids

**Figure 1:**
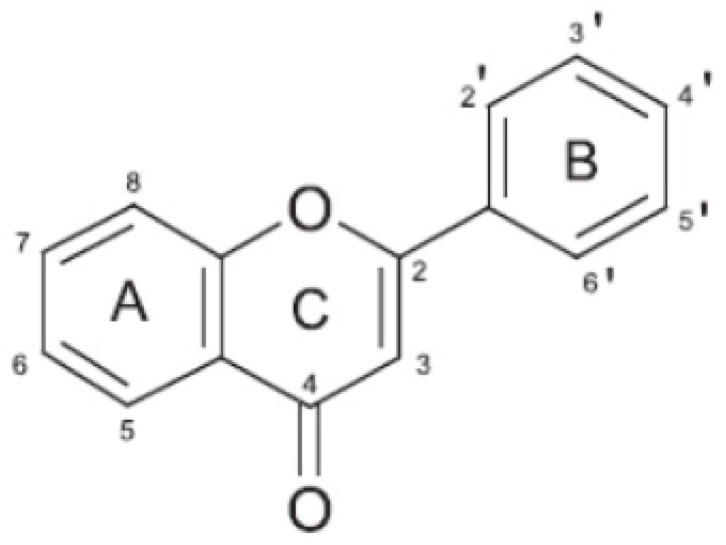
Basic chemical structure of *Citrus* flavonoids.

Flavonoids are pigments commonly present in the genus *Citrus* that are responsible for flower and fruit color. They are low molecular weight polyphenolic compounds, widely found in the plant kingdom as secondary metabolites. They are characterized by a common C6-C3-C6 structure consisting of two benzene rings (A and B) linked through a heterocyclic pyran ring (C) (Figure 1).

Flavonoids containing an hydroxyl group in position C-3 of the C ring are classified as 3-hydroxyflavonoids (flavonols, anthocyanidins, leucoanthocyanidins, and catechins), and those lacking it as 3-desoxyflavonoids (flavanones and flavones). At present, more than 9000 flavonoids have been characterized, some of which are clinically used. The large number of compounds arises from various combinations of multiple hydroxyl and methoxyl groups substituting the basic flavonoids skeleton. Flavonoids are divided into six classes on the basis of their chemical structures: flavones, flavanones, flavonols, isoflavones, anthocyanidins, and flavans. Flavonoids are mainly present in plants as glycosides, while aglycones (the forms lacking sugar moieties) occur less frequently. Therefore, a large number of flavonoids result from many different combinations of aglycones and sugars, among which mainly d-glucose and l-rhamnose bound to the hydroxyl group at the C-3 or C-7 position.

**Figure 2:**
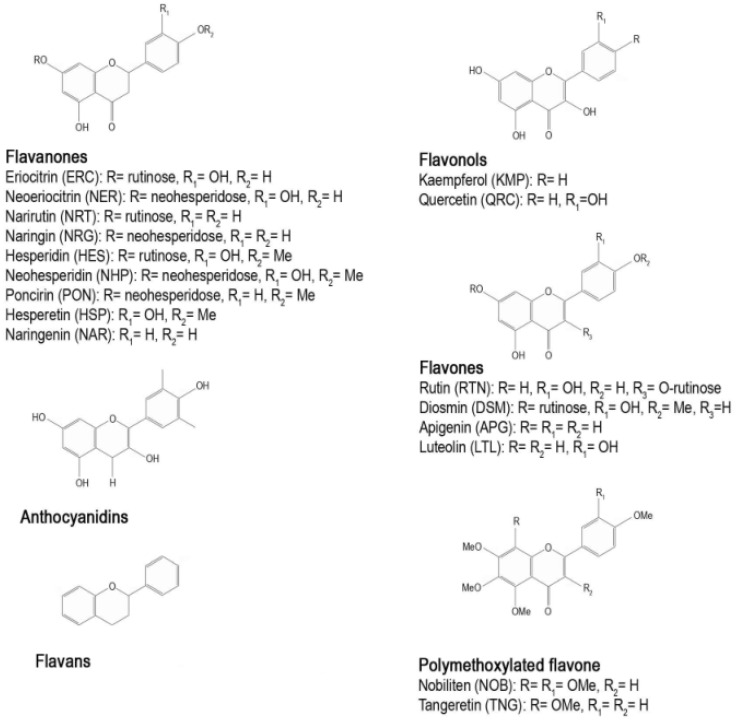
Structural formula of some flavonoids isolated from *Citrus* fruits and their chemical substituents.

More than sixty types of flavonoids have been identified in CF: flavanones are the flavonoids most widely present, followed by flavones, flavonols, and anthocyanins (the latter only in blood oranges). Some flavonoids, such as hesperidin, naringin, and polymethoxylated flavones (PMFs) are characteristic compounds contained in *Citrus* while others like rutin and quercetin are common throughout the plant kingdom [13]. Figure 2 shows the main structural formula of some flavonoids isolated from CF, and their chemical substituents.

**Figure 3:**
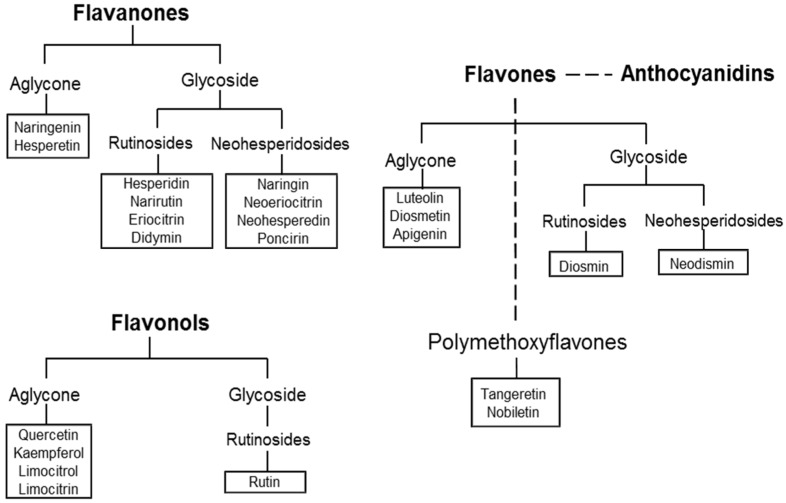
Classification of *Citrus* flavonoids.

Flavanones (2,3-dihydro-2-phenylchromen-4-one) occur almost exclusively in CF and are present in both the glycoside or aglycone forms (Figure 3). Naringenin and hesperetin are the most important flavanones present in aglycone forms, while the glycosidic forms are grouped into two types: neohesperidosides and rutinosides. Glycosylation occurs at position 7, either by rutinose or neohesperidose, disaccharides formed by a glucose and a rhamnose molecule differing only in the type of linkage (1 → 6 or 1 → 2). Naringin, neoeriocitrin, neohesperedin, and poncirin consist of a flavanone with neohesperidose (rhamnosyl-α-1,2 glucose), and they have a bitter taste; while hesperidin, narirutin, eriocitrin, and didymin consist of a flavanone with rutinose (rhamnosyl-α-1,6 glucose), and have no taste. Flavanones, usually present in diglycoside form, give CF their characteristic taste.

Flavonols (3-hydroxy-2-phenylchromen-4-one) may be considered to be the 3-hydroxy derivatives of flavones. Glycosylation occurs preferentially at the 3-hydroxyl group of the central ring, and the predominant types are 3-*O*-monoglycosides. The most common flavonol aglycones are quercetin and kaempferol, while rutin and rutinosides are the main glycosidic forms.

The most abundant flavones (2-phenylchromen-4-one) present in the aglycone form are luteolin, diosmetin, and apigenin, while diosmin and neodismin represent the principal flavones present in the rutinoside and neohesperidoside forms, respectively. The PMFs tangeretin and nobiletin are present in smaller quantities.

Anthocyanins (2-phenylchromenylium), are metabolites of flavones structurally derived from pyran or flavan. In CF, they are present only in blood oranges. Anthocyanidins are anthocyanins with a sugar group, in which glycosylation with glucose, arabinose, or galactose almost always occurs at the 3-position.

The most abundant *Citrus* flavonoids are flavanones, e.g., hesperidin, naringin, or neohesperidin. However, there are flavones, e.g., diosmin, apigenin, or luteolin, that generally display higher biological activity, despite occuring in much lower concentrations. Of note are apigenin, which has shown particularly good anti-inflammatory activity, and diosmin and rutin that are important venotonic agents present in several pharmaceutical products. The beneficial effects of flavonoids are mainly due to their anti-oxidant properties which can play a key role in fighting several degenerative diseases. However, there is recent increasing evidence linking the pharmacological activity of *Citrus* flavonoids to their ability to inhibit the activity of intracellular signaling molecules, such as phosphodiesterases, kinases, topoisomerases, and other regulatory enzymes [14]. Blocking protein kinases and lipid-dependent signaling cascades results in alterations in the phosphorylation state of target molecules, with the consequent modulation of gene expression implicated in many degenerative diseases including cancer. Many studies designed to uncover a structure–activity relationship have demonstrated that anti-oxidant, enzyme-inhibitory, or anti-proliferative activities of some flavonoids are dependent upon particular structural factors. The structure oxidation state (flavanone, flavone, etc.), substituents (position, number, and nature of groups in both the A and B rings of the flavonoid structure), and the presence of glycosylation may be important determinant features of flavonoid activity [15,16]. More specifically, studies on melanoma cell lines using several flavonoids of *Citrus* origin have shown the presence of the C2–C3 double bond on the C ring, conjugated with the 4-oxo function, to be critical for this biological activity [17]. Moreover, the presence of three or more hydroxyls in any of the rings of the flavonoid skeleton significantly increased the anti-proliferative activity observed in melanoma B16-F10 cell cultures [18].

## 3. Preclinical Studies

Carcinogenesis is a multi-step process of genetic and epigenetic alterations leading to the progressive transformation of normal cells towards malignancy. The process of carcinogenesis can be divided into three main stages: (i) initiation, a phase in which cellular exposition to a carcinogenic agent leads to irreversible alterations, usually at the DNA level. In this phase cells react to carcinogens by the activation of enzymes involved in the metabolism of xenobiotics that, while aiming to inactivate, may generate a mutagenic compound responsible for DNA damage and mutations, thereby initiating cancer development; (ii) the tumor promotion stage is characterized by the proliferation of abnormal cells that may initiate a pre-neoplastic focus. In this phase over-activation and/or over-expression of enzymes involved in the synthesis of nucleotides and DNA (e.g., ornithine decarboxylase), as well as in the regulation of the differentiation process (DNA polymerase or topoisomerases) occur. Moreover, oxidative stress caused by the overproduction of reactive oxygen species (ROS) induces further cell damage and genome instability; (iii) progression is the final stage of carcinogenesis. It is characterized by an uncontrolled proliferation of tumor cells which also acquire the ability to invade neighboring tissues and to form metastasis at distant sites, coupled with a loss of capacity for apoptosis or senescence. Hence, metastasis is the spread of cancer cells from a primary tumor to distant sites in the cancer patient’s body. Angiogenesis is the first step of the metastatic process that leads to the formation of new blood capillaries by outgrowth or sprouting of pre-existing blood vessels. It allows the tumor to be fed and facilitates the access of tumor cells to the bloodstream. Indeed, tumor vessels are more permeable than normal ones, since tumor-associated endothelial cells are enlarged and loosely connected. Therefore, the metastatic process is the end result of a complex series of events depending on the ability of tumor cells to detach from the primary tumor, migrate, and invade connective tissues, entering the vascular or lymphatic system, through which vital organs are reached where they proliferate to form a distant metastasis. The tendency of a primary tumor to form metastasis is the hallmark of malignant cancer, and has important diagnostic, prognostic, and therapeutic implications.

Interest in nutrition and cancer has grown considerably, as evidenced by the rapid proliferation of studies examining nutritional exposure in relation to cancer risk [19]. A large body of in vitro and in vivo studies have shown that fruits and vegetables may have an important role in the maintenance of a healthy lifestyle and the reduction of cancer risk. Their potential health benefits are probably due to the presence of secondary metabolites ubiquitous in the plant kingdom that are considered non-nutritional but which are essential for the maintenance of health. Thus, in the last decade, bioactive compounds including flavonoids, carotenoids, ascorbic acid, and limonoids have been intensively investigated for their potential antioxidant, anti-inflammatory, and anti-cancer activities. Several compounds are responsible for *Citrus* antitumoral effects; of these, vitamin C is considered an important micronutrient through which CF exert their antioxidant effects by trapping free radicals and reactive oxygen molecules, thus protecting against oxidative damage, inhibiting the formation of carcinogens and protecting DNA from damage [20]. Flavonoids also exhibit antioxidant and free radical scavenging properties, interfering with the oxidative/anti-oxidative potential of the cell [21]. Furthermore, there are numerous reports showing flavonoids to be able to act at various stages of carcinogenesis, and specifically to interact with proteins involved in cancer development.

Growing experimental evidence supports the view that *Citrus* flavonoids exert their anti-cancer effects through a number of different mechanisms. They may act as suppressing agents, preventing the formation of new cancers from pro-carcinogens or as blocking agents, disenabling carcinogens from achieving initiation, as well as preventing the onset of the tumor promotion stage. Moreover, *Citrus* flavonoids may function as transformation agents, facilitating the biotransformation of carcinogens into inactive metabolites. Finally, they behave as both anti-angiogenic and anti-metastatic agents, preventing the formation of new vessels and metastasis [14,22]. Table 1 shows the principal cancer-related processes modulated by *Citrus* flavonoids.

### 3.1. Initiation Phase Inhibition by *Citrus* Flavonoids

In the last twenty years, there has been an increasing awareness that flavonoids and other naturally-occurring substances in plants have protective effects against environmental mutagens/carcinogens and endogenous mutagens [23]. In support of this, there are numerous experimental findings suggesting that certain *Citrus* flavonoids may exert preventive effects against DNA damage induced by a variety of carcinogens [24]. Naringenin and rutin prevent the accumulation of ultraviolet radiation-B (UV-B)-induced DNA damage [25] by a mechanism that may involve the ability of flavonoids to neutralize free radicals generated near DNA, promoting mutations. The radical scavenging property of flavonoids is also responsible for quercetin protective effect against mercury-induced DNA damage and oxidative stress in a human-derived liver cell line (HepG2), that seems to be due to the maintenance of redox status [26]. Moreover, it has been observed that naringenin at low doses (10–80 μM) can stimulate DNA repair following oxidative damage in a human lymph node prostate cancer cell line (LNCaP), leading to a significant increase in the levels of several major enzymes in the DNA base excision repair pathway [27]. In in vivo experiments, naringenin has demonstrated its capability to inhibit *N*-diethylnitrosamine (NDEA)-induced hepatocarcinogenesis [28,29]. Naringin has been found to reduce the rate of micronuclei formed by ifosfamide in mouse blood cells [30] and to exert protective action against DNA deterioration induced by daunorubicin in mouse hepatocytes and cardiocytes, suggesting that this flavonoid may be useful in reducing the adverse effects found in anthracycline treatments [31]. Moreover, it accelerated the regression of pre-neoplastic lesions in rats exposed to 1,2-dimethylhydrazine (DMH) [32]. Experiments performed using in vivo models of genotoxicity induced by cyclophosphamide show that the antioxidative activity of hesperidin (100, 200, and 400 mg/kg body weight (BW) administered by gavages for five consecutive days) may reduce the frequency of micronucleated polychromatic erythrocytes (MnPCEs) induced by chemotherapy drugs [33]. Furthermore, in the presence of a mammalian metabolic activation system, naringin, apigenin, hesperetin, and other flavonoids (300 μg/plate) have been shown to produce antimutagenic effects against aflatoxin B1 (1 μg/plate), with an inhibition rate of more than a 70% in *Salmonella typhimurium*. In this study, the structure–activity relationship analysis suggests the flavonoid configuration containing the free 5-, 7-hydroxyl group to be essential [34].

Flavonoids may also inhibit the first phase of carcinogenesis through an increase in detoxification processes by modulating enzyme activity resulting in the decreased carcinogenicity of xenobiotics. For example, naringenin inhibits the activity of aromatase (CYP19) in Chinese hamster ovary (CHO) cells, thereby decreasing estrogen biosynthesis and inducing antiestrogenic effects, which are important in breast and prostate cancers [35]. Quercetin has instead proven to be a potent non-competitive inhibitor of sulfotransferase 1A1, suggesting a role for potential chemopreventive agents in sulfation-induced carcinogenesis [36]. The chemopreventive potential of diosmin, naringenin, naringin, and rutin against CYPlA2-mediated mutagenesis of heterocyclic amines produced by high temperature cooking of meat was hinted by Bear and Teel [37]. Several reports have described the potential anti-mutagenic properties of apigenin. For instance, exposure to apigenin prior to a carcinogenic insult has been shown to offer a protective effect in both murine skin and colon cancer models [38], as well as to prevent the genotoxic effects of benzo(α)pyrene (BP) in vivo. Indeed, Khan et al. [39] demonstrated that apigenin (2.5 and 5 mg/kg orally) reverts BP-induced depletion in the levels of glutathione (GSH), quinone reductase (QR), and glutathione-*S*-transferase (GST), while also reducing DNA strand breaks and damage. Increased GSH by apigenin also enhances endogenous defense against oxidative stress [40]. Moreover, topical application of apigenin has been proven to reduce dimethyl benzanthracene-induced skin tumors by strongly inhibiting epidermal ornithine decarboxylase, an enzyme that plays a key role in tumor promotion [41]. In addition, apigenin administration has been reported diminish the incidence of UV light-induced cancers and to increase tumor-free survival in vivo [42]. Moreover, apigenin as well naringenin, suppress colon carcinogenesis in azoxymethane (AOM)-treated rats [43].

The antigenotoxic activity of hesperidin was investigated by Nandakumar et al., [44]. They reported that daily administration of hesperidin at a concentration of 30 mg/kg BW for 45 days prevented 7,12-dimethylbenz(α)anthracene (DMBA)-induced experimental breast cancer formation, presumably by the regulation of both phase I and phase II metabolizing enzymes, and through its strong antioxidant activity. The results also revealed that the flavanone may act both by modulating the energy reservoir of the cell and by maintaining oxidative phosphorylation. Also, the aglycone hesperetin has been reported to modulate xenobiotic-metabolizing enzymes during DMH-induced colon carcinogenesis [45]. Tangeretin, a pentamethoxy flavone present in significant amounts in CF peel, was found to suppress DMBA-induced breast cancer in rats [46].

Chronic inflammation is closely connected to the carcinogenic process. Indeed, nobiletin has been shown to inhibit DMBA/tetradecanoyl-13-phorbol acetate (TPA)-induced skin tumor formation by reducing the number of tumors per mouse, manifesting its potential in inflammation-associated tumorigenesis [47]. The studies discussed above are summarized in Table 2.

### 3.2. Inhibition of Tumor Development

A great number of in vitro studies have demonstrated that *Citrus* flavonoids reduce the growth of several types of tumor cells in cultures. Tangeretin, nobiletin, quercetin and taxifolin have anti-proliferative effects on squamous cell carcinoma HTB43 [48], as well as on many other tumoral cell lines. Tangeretin, a PMF present mainly in the peel of tangerine and other CF, induced apoptosis in human myeloid leukaemia HL-60 cells, without causing cytotoxicity in human peripheral blood mononuclear cells [49,50]. Tangeretin and nobiletin (another PMF widely found in the mandarin epicarp) also inhibited the proliferation of both human breast cancer cell lines (MDA-MB-435 and MCF-7) and a human colon cancer cell line (HT-29) in a concentration- and time-dependent manner, by blocking cell cycle progression at the G1 phase without inducing cell death [51]. This study showed tangeretin IC_50_ values of 30–40 μM for breast and colon cell lines, and slightly higher values for nobiletin, while in other reports tangeretin exhibited much greater potency [49,50]. However, this discrepancy could be caused by differences related to both cell type and experimental procedures. The inhibition of the activity of cyclin-dependent kinases 2 (Cdk2) and 4 (Cdk4), accompanied by an increase in Cdk inhibitors p21 and p27 seems to be the mechanism through which tangeretin arrests cell cycle progression at the G1 phase in colon adenocarcinoma COLO 205 cells [52]. Yoshimizu et al. [53] documented the growth-inhibitory action of nobiletin, both alone and in combination with cisplatin, in various human gastric cancer cell lines (TMK-1, MKN-45, MKN-74, and KATO-III), through the induction of apoptosis and cell cycle deregulation. Interestingly, orange peel extract (OPE) containing 30% polymethoxyflavones, such as tangeretin (19.0%), heptamethoxyflavone (15.24%), tetramethoxyflavone (13.6%), nobiletin (12.49%), hexamethoxyflavone (11.06%) and sinensitin (9.16%), inhibited tumorigenesis in Apc^(Min/+)^ mice by increasing apoptosis [54]. OPE also decreased the development of hyperplastic lesions in mouse mammary glands [55]. The reduction of mammary cancer cell growth caused by tangeretin may be related to the inhibition of mitogen-activated protein kinase (MAPK)/extracellular-signal-regulated kinase (ERK) phosphorylation and of other proteins like adducin α and γ, protein kinase Cδ, signal transducer and activator of transcription (STAT) 1 and 3, and stress-activated protein kinase (JNK) [56]. Tangeretin and nobiletin also inhibited the proliferation of both SH-SY5Y neuroblastoma cells [57] and brain tumor cells [58], reducing also invasion, migration, and adhesive properties. Moreover, it has been reported that tangeretin sensitizes cisplatin-resistant human ovarian cancer cells through the downregulation of the phosphoinositide 3-kinase (PI3K)/protein kinase B (also known as Akt) signaling pathway, suggesting a potential approach for the treatment of drug-resistant cancers [59]. Tangeretin also induced apoptosis in gastric cancer AGS cells through the activation of both extrinsic and intrinsic signaling pathways [60]. Nobiletin and the coumarin auraptene have been reported to counteract prostate carcinogenesis both in vitro and in vivo. In particular, nobiletin inhibited the growth of several prostate cancer cell lines with IC_50_ values of around 100 μM, by a mechanism involving apoptosis and cell cycle arrest at the G_0_/G_1_ phase, as well as inhibited development of prostate adenocarcinomas in a transgenic rat model [61]. The preventive effects of nobiletin on prostate cancer have recently been confirmed in a study that also reported the ability of this flavonoid to reduce the risk of colon cancer [62]. Furthermore, nobiletin reduces AOM-induced rat colon carcinogenesis [63] and, like quercetin (100 ppm), is able to decrease preneoplastic lesions and serum levels of both leptin and insulin in an in vivo model of colon carcinogenesis, suggesting a promising role in preventing tumors associated with obesity [64,65]. Experiments performed using both in vitro and in vivo models showed the anti-proliferative property of nobiletin on lung cancer cells. The mechanism involves the activation of the apoptotic process and cell cycle arrest at the G2/M phase due to decreased Bcl-2 and increased Bax protein expression, both of which positively correlated with elevated expression of p53 [66]. As reported by Ohnishi et al. [67], nobiletin treatment suppressed HepG2 and MH1C1 hepatocarcinoma cell growth by inducing cell cycle inhibition and apoptosis, but without apparent effects in the early stages of in vivo hepatocarcinogenesis. In glioma cells, it suppresses proliferation by inhibiting Ras activity and mitogen-activated protein/extracellular signal-regulated kinase (MEK/ERK) signaling cascade, probably via a Ca^2+^-sensitive protein kinase C (PKC)-dependent mechanism [68]. There are more recent results that demonstrate the ability of nobiletin to inhibit cell growth and migration via cell-cycle arrest and suppression of the MAPK and Akt pathways [69]. In human gastric p53-mutated SNU-16 cells, nobiletin was found to be effective in inhibiting cell proliferation, inducing apoptosis, and enhancing the efficacy of 5-Fluorouracil (FU) [70]. Its anti-cancer effects have also been demonstrated in acute myeloid leukemia cells [71], where it was responsible for the induction of cell-cycle arrest and apoptosis. Moreover, orally administrated nobiletin inhibited colitis-associated colon carcinogenesis in AOM/dextran sulfate sodium-treated mice [72].

Apigenin is a flavone present mainly in fruits and vegetables, and among *Citrus* species it is abundant in grapefruit. It possesses anti-inflammatory and free radical scavenging activity, and as a candidate anti-cancer agent, is capable of reducing cancer cell proliferation of without affecting normal cells. It has been reported that apigenin possesses growth inhibitory properties in breast cancer, inducing apoptosis by: (i) the involvement of the caspase cascade [73]; (ii) inhibiting STAT3 and nuclear factor kappa B (NF-κB) signaling in HER2-overexpressing breast cancer cells [74]; (iii) reducing the activity of both PI3K and Akt kinase [75] and regulating the p14ARF-Mdm2-p53 pathway [76]. Apigenin is reported to exert growth inhibitory effects by increasing the stability of p53, leading to cell cycle arrest in many cancer cell lines, including rat neural and liver epithelial cells, as well as human breast, ovarian, cervical, prostate, colon, and thyroid cancers [77]. In epidermal cells and fibroblasts reversible G2/M and G0/G1 arrest is also mediated by the inhibition of p34 (Cdc2) kinase activity [78,79], while in breast carcinoma the G2/M phase cell cycle arrest after apigenin treatment led to a significant decrease in cyclins (B1, D1, and A) and cyclin-dependent kinase (Cdk1 and 4) protein levels [80]. In pancreatic cancer cell lines, apigenin caused both time- and concentration-dependent inhibition of DNA synthesis and cell proliferation through G2/M phase cell cycle arrest caused by the suppression of cyclin B-associated Cdc2 activity [81,82]. Moreover, in the same cell lines, it inhibited the glycogen synthase kinase-3β/NF-ĸB signaling pathway and upregulated the expression of cytokine genes, which potentially contributed to its anti-cancer properties [83]. In addition, apigenin has been shown to induce WAF1/p21 levels, resulting in G1 phase cell cycle arrest in androgen-responsive (LNCaP) and androgen-refractory (DU145) human prostate cancer cells [84,85]. Indeed, the apoptosis observed in these cell lines appeared to be correlated with: (i) the alteration in Bax/Bcl-2 ratio; (ii) the down- regulation of the constitutive expression of NF-ĸB/p65; (iii) the release of cytochrome c; (iv) the induction of apoptotic protease activating factor-1 (Apaf-1), which leads to caspase activation and PARP-cleavage [84,85]. Apigenin-induced growth inhibition by different mechanisms has also been reported in colon [86,87], prostate [88], and neuroblastoma [89,90] cancer cells. In endothelial cells, the anti-proliferative effect exerted by the flavanone is due to the blocking of cells in the G2/M phase, as a result of the accumulation of the hyperphosphorylated form of retinoblastoma protein [91]. Diosmin, another important *Citrus* flavone (mostly due to its venotonic activity), occurs naturally as a glycoside, and after ingestion is rapidly transformed by intestinal flora to its aglycone form, diosmetin. Diosmin has been shown to inhibit Caco-2 and HT-29 colon cancer cell growth [92]. In the hepatocellular carcinoma HA22T cells, it inhibited cell viability, reduced cellular proliferative proteins, and induced cell cycle arrest in the G2/M phase through p53 activation and inhibition of the PI3K-Akt-mouse double minute 2 homolog (MDM2) signaling pathway. In addition, it suppressed tumor growth through protein phosphatase 2 (PP2A) activation in HA22T-implanted xeno-graft nude activation [93]. The effectiveness of diosmin as an anti-cancer agent has also been demonstrated in DU145 prostate cancer cells, where it promotes genotoxic events and apoptotic cell death [94]. Moreover, it has been shown that diosmin may reduce the development of esophageal cancer induced by *N*-methyl-*N*-amylnitrosamine (MNAN) when given during the initiation phase [95], decreases oral carcinogenesis initiated by 4-nitroquinoline 1-oxide (4-NQO) [96], counteracts *N*-butyl-*N*-(4-hydroxybutyl)nitrosamine (OH-BBN)-induced urinary-bladder carcinogenesis [97], and prevents AOM-induced rat colon carcinogenesis, either alone or in combination with hesperidin [98]. In these cases [95,96,97,98], rats were fed a diet containing diosmin (1000 ppm), hesperidin (1000 ppm), or diosmin + hesperidin (900 ppm and 100 ppm, respectively), and the cancer inhibition found could be related to the suppression of the increased cell proliferation caused by the carcinogens in the affected mucous membranes.

Quercetin is a water-soluble flavonol, widely distributed in nature and the most common dietary flavonol. It represents the aglycone form of a number of other flavonoid glycosides, such as rutin and quercitin. In CFit is present mainly in lemon peel. Experimental data have shown quercetin to be a potential anti-carcinogenic agent against several human tumor cell lines, including HL-60 (promyelocytic leukemia cells), A431 (epithelial carcinoma cell line), SK-OV-3 (ovary adenocarcinoma), HeLa (cervical carcinoma) and HOS (osteosarcoma) [99]. The inhibitory effect of quercetin on HL-60 growth may be due to the induction of apoptosis mediated by an up-regulation of pro-apoptotic Bax and post-translational modification (phosphorylation) of anti-apoptotic Bcl2 [100]. This flavonol also demonstrated concentration-dependent anti-proliferative activity against both meningioma [101] and colon cancer cells (CRC) [102]. Growth inhibition of several CRC cells has been reported and numerous mechanisms explaining the in vitro anti-proliferative effect of quercetin have been proposed [103]. Interestingly the combination of quercetin and low-frequency ultrasound selectively induced cytotoxicity in skin and prostate cancer cells, while having minimal effect on corresponding normal cell lines [104]. Quercetin has been reported to induce cell growth inhibition in MDA-MB-231 breast cancer cells by inhibition of the F-box protein S-phase kinase-associated protein 2 (Skp2) and induction of p27 expression, thereby blocking cell cycle progression [105]. Moreover, several reports have shown that if quercetin is associated with antineoplastic drugs it may then play a relevant role in development of chemotherapeutic combinations. For example, in human breast cancer cells, quercetin inhibits lapatinib-sensitive and -resistant breast cancer cell growth by modifying levels of factors that regulate cell cycle G2/M progression and apoptosis, such as cyclin B1, p-Cdc25c (Ser216), Chk1, caspase 3, caspase 7, and PARP [106]. In breast cancer cells, it potentiated the antitumor effects of doxorubicin, attenuating unwanted cytotoxicity to non-tumoral cells [107], and markedly increased the effect of adriamycin in a multidrug-resistant MCF-7 human breast cancer cell line [108] and in MCT-15 human colon carcinoma cells [109].

Naringin and naringenin are two of the most abundant flavanones in CF, although the amounts differ. Naringenin is the aglycone and is a metabolite of naringin (naringenin-7-neohesperoside), the main flavonoid of grapefruit. Diverse biological and pharmacological properties, including anti-carcinogenic activity, have been reported for both of these flavanones. Kanno et al. [110] showed the anti-proliferative effect of naringenin in a range of human cancer cell lines (breast, stomach, liver, cervix, pancreas, and colon) as well as its ability to inhibit tumor growth in sarcoma S-180-implanted mice. The same authors reported that the exposure of human promyeloleukemia HL-60 cells to naringenin at concentrations up to 0.5 mM induced apoptosis via the activation of NF-κB, while a higher concentration (1 mM) reduced intracellular ATP levels, causing mitochondrial dysfunctions leading to necrosis [111]. Naringenin-induced inhibition of colon cancer cell proliferation has also been reported by Frydoonfar et al. [112]. A mechanism through which naringenin might cause a reduction of breast cancer growth seems to be the impairment of glucose uptake. Indeed, in MCF-7 cells, the flavanone impaired the insulin-stimulated glucose uptake, thus decreasing the availability of glucose concentration in the culture medium and inhibiting proliferation [113]. In human leukemia THP-1 cells, naringenin exerts an anti-proliferative effect in a concentration-dependent manner, inducing apoptosis through the modulation of the Bcl-2 family, mitochondrial dysfunction, activation of caspases, and PARP degradation that correlate with inactivation of the PI3K/Akt pathway [114]. Using the same cell line, Shi et al. [115] have demonstrated naringenin may enhance curcumin-induced apoptosis through inhibition of the Akt and ERK pathways, and by activating the JNK and p53 pathways. In human epidermoid carcinoma A431 cells, the ability of naringenin to induce apoptotic cascade and cell cycle arrest in the G0/G1 phase has been demonstrated [116]. Several in vitro studies have demonstrated the naringenin-induced intrinsic apoptotic pathway initiated by the caspase cascade [111,114,117]. It has also been reported activation of the apoptosis extrinsic pathway, triggered by ligands binding plasma membrane death receptors. Indeed, it has been observed that naringenin enhances tumor necrosis factor-related apoptosis-inducing ligand (TRAIL)-induced apoptosis in TRAIL-resistant A549 human lung cancer cells by the upregulation of TRAIL receptor 5 (death receptor 5, DR5, also named TRAIL-R2)) without inhibition of cell growth in human normal lung fibroblast WI-38 cells [118]. Moreover, naringenin (50 μM) and other flavonoids, among which hesperetin and apigenin, produced a more than three-fold increase in mitoxantrone accumulation by inhibition of breast cancer resistance protein (BCRP; an ATP-binding cassette transporter conferring multidrug resistance to a number of important anti-cancer agents) in BCRP-overexpressing MCF-7 (breast cancer) and NCI-H460 (lung cancer) cells, whereas the glycoside form (naringin) had no significant effects [119]. The presence of the 2,3-double bond in the C ring of flavonoids, as well as ring B being attached at position 2, hydroxylation at position 5, lack of hydroxylation at position 3, and hydrophobic substitution at positions 6, 7, 8, or 40, are structural properties important for potent flavonoid–BCRP interaction, and critical for potent BCRP inhibition [120]. Some studies have suggested that naringenin also inhibits the P-glycoprotein (P-gp), thus improving antitumor activity both in vitro [121] and in vivo [122,123]. Conversely, other experimental studies indicate that naringenin modulates drug efflux pathways by inhibiting the activity of multidrug resistance-associated proteins (MRPs) but not P-gp [124]. Similarly, Zhang and collaborators [124] have claimed that doxorubicin in combination with naringenin enhanced antitumor activity in vivo, while others have asserted that the pharmacokinetics of intravenously administered doxorubicin (the plasma concentration, biliary, and urinary clearance and tissue distribution) is not altered by pre-treatment with naringin, naringenin, and quercetin [125]. A number of in vivo studies on the antitumor effects of naringenin have also been performed. These found that it suppresses colon carcinogenesis through the aberrant crypt stage in AOM-treated rats [43], reduces tumor size and weight loss in *N*-methyl-*N*’-nitro-*N*-nitrosoguanidine-induced gastric carcinogenesis [126,127], promotes apoptosis in cerebrally-implanted C6 glioma cells rat model [128] and, like naringin, inhibits oral carcinogenesis [129].

Several findings have identified naringin to be a promising chemotherapeutic agent for diverse types of cancers. Naringin (750 μM) showed an anti-proliferative effect on SiHa human cervical cancer cells through cell cycle arrest in the G2/M phase and apoptosis induction via disruption of mitochondrial transmembrane potential, and the activation of both the intrinsic and extrinsic pathways [130]. By contrast, naringin (1 mM) induced growth inhibition and apoptosis by suppressing the NF-κB/COX-2-caspase-1 pathway on HeLa cells [131]. Recently, the role of glycoconjugates in cancer cells has been a focus because of their regulatory effects on malignant phenotypes. A study by Yoshinaga [132] reported naringin to suppress HeLa and A549 cell growth through the alteration of glycolipids. This effect may largely be due to the attenuation of epidermal growth factor receptor (EGFR) signaling through GM3 ganglioside accumulation. Triple-negative (ER-/PR-/HER2-) breast cancer is an aggressive cancer with poor prognosis and a lack of targeted therapies. In this kind of tumor, Li et al. [133] demonstrated that naringin inhibited cell proliferation and promoted cell apoptosis and G1 cycle arrest. These effects were accompanied by increased p21 levels and decreased survival by modulation of the β-catenin pathway.

Moreover, 100 μM naringin resulted in a significant concentration-dependent growth inhibition of 5637 bladder cancer cells together with of cell-cycle blocking [134]. In this cell line, the naringin-induced anti-proliferative effect seems to be linked to the activation of Ras/Raf/ERK-mediated p21WAF1 induction, which in turn leads to a decrease in the levels of cyclin D1/CDK4 and cyclin E-CDK2 complexes, causing G1-phase cell-cycle arrest [134]. Recently, naringin has been investigated regarding its ability to induce autophagy. Several studies have reported that autophagy promotes cancer cell death in response to various anti-cancer agents on apoptosis-defective cells [135,136]. Accordingly, over-activation of autophagy in cancer cells has been proposed to be an important death mechanism occurring in the tumor progression phase, where apoptosis is limited [136]. In AGS gastric adenocarcinoma cells, naringin showed autophagy-mediated growth inhibition by suppressing the PI3K/Akt/mTOR cascade through MAPKs activation [137]. Naringin has been demonstrated to reduce glioblastoma cell proliferation by inhibiting the FAK/cyclin D1 pathway, and promoting cell apoptosis by influencing the FAK/bads pathway [138].

Furthermore, an in vivo study documented that grapefruit pulp powder (13.7 g/kg) or isolated naringin (200 mg/kg) or limonin (200 mg/kg) protect against AOM-induced aberrant crypt foci (ACF) by suppressing proliferation and elevating apoptosis through anti-inflammatory activities, suggesting that the consumption of grapefruit or its flavonoids may help to suppress colon cancer development [139]. Camargo et al. [140] showed that the treatment of rats bearing Walker 256 carcinosarcoma (W256) with 25 mg/kg of naringin reduced tumor necrosis factor-α (TNF-α) and interleukin-6 (IL-6) levels and tumor growth by ~75%. Very recently, it has been proven that naringin prevent intestinal tumorigenesis in a adenomatous polyposis coli multiple intestinal neoplasia (Apc^(Min/+)^) mouse model [141].

Another important *Citrus* flavanone is hesperidin (hesperetin-7-rutinoside), the principal flavonoid in sweet orange and lemon, being the glycosides form of hesperetin (free state). It is water-soluble as a glycoside conjugate due to the presence of the sugar in its structure, which on ingestion releases its aglycone hesperetin. Along with other flavonoid compounds, hesperidin has been widely reported to possess venotonic and vasculo-protective pharmacological properties, and it is effectively used as a supplement in patients suffering from blood vessel disorders including capillary fragility and excessive permeability [142]. Both hesperidin and hesperetin have shown anti-cancer activities, although the latter exhibited higher anti-proliferative activity in vitro. Chen et al. [143] showed hesperetin to exert stronger cytotoxic activity than hesperidin in the HL-60 human leukemia cell line. Moreover, at the same concentrations (40 and 80 μM), hesperetin induced apoptosis, while hesperidin did not. The Authors suggest that the rutinoside group at C-7 causes the reduction of apoptotic induction on HL-60 cells by hesperidin. This hypothesis is strengthened by evidence that the aglycone naringenin also induces anti-proliferative and pro-apoptotic effects, but not the glycone naringin. Furthermore, hesperetin inhibits the expression of CDK2, CDK4, and cyclin D, thus inducing cell cycle arrest in the G1 phase, which in turn reduces MCF-7 cell proliferation in a concentration-dependent manner [144]. Moreover, hesperetin (5 to 100 μM) inhibits human colon adenocarcinoma HT-29 cellular growth and induces apoptosis via the Bax-dependent mitochondrial pathway, involving oxidant/antioxidant imbalance [145]. It also enhances Notch1 levels, that in turn decreases the expression of the neuroendocrine tumor markers ASCL1 and CgA, causing inhibition of human gastrointestinal carcinoid (BON) cell growth [146]. Furthermore, hesperetin exerts anti-proliferative and pro-apoptotic effects in human cervical cancer SiHa cells, via both death receptor- and mitochondria-related mechanisms [147], while it induces ROS-mediated cell death in hepatocarcinoma cells [148]. In the same study, the Authors showed that hesperetin significantly inhibited the growth of xenograft tumors [148]. Hesperidin (20 mg/kg BW) suppressed cell proliferation markers, angiogenic growth factors, COX-2 mRNA expression, enhanced apoptosis, and reduced aberrant crypt foci in DMH-induced colon carcinogenesis in rats [149,150].

Anti-proliferative activity has also been described for the glycone hesperidin: Patil et al. [151] found that it inhibits cell cycle progression in Panc-28 human pancreatic carcinoma cells, while Park et al. [152] described its cytotoxic and pro-apoptotic effects on SNU-C4 human colon cancer cells. In HepG2 hepatocarcinoma cells, its ability to induce apoptosis via both mitochondrial and death receptor pathways has been demonstrated [153], as well as the non-apoptotic programmed cell death namely paraptosis [154]. Hesperidin also inhibits proliferation of Ramos Burkitt’s lymphoma cells and sensitizes them to doxorubicin-induced apoptosis through the inhibition of both constitutive and doxorubicin-mediated NF-κB activation in a PPARγ-independent manner [155]. In hematopoietic malignancies, hesperidin promoted p53 accumulation and downregulated constitutive NF-κB activity in both PPARγ-dependent and -independent pathways [156]. Induction of apoptosis by hesperidin has also been reported in human mammary carcinoma MCF-7 [157,158] and human cervical cancer HeLa cells [159].

Other reports have shown that hesperidin and neohesperidin increase the sensitivity of Caco-2 cells to doxorubicin, which is consistent with decreased Pgp activity demonstrated in drug-resistant human leukaemia cells (CEM/ADR5000) at non-toxic concentrations (0.32–32 μM) [160]. Inhibition of Pgp has also been described for hesperetin and quercetin in breast cancer resistance protein (BCRP/ABCG2)-overexpressing cell lines [161]. Moreover, hesperidin has been reported suppress proliferation of both human breast cancer and androgen-dependent prostate cancer cells through mechanisms other than antimitotic ones, suggesting a possible interaction with androgenic receptors [162].

Encouraging results in vivo of carcinogenesis inhibition by hesperidin have also been observed. The compound (500 ppm/kg BW) was found to inhibit 4-NQO-induced oral carcinogenesis and to decrease the number of lesions, polyamine levels in tongue tissue, and cell proliferation activity [163]. Later, the same group reported the inhibition of 4-NQO, AOM, MNAN, and OH-BBN-initiated tumorigenesis by hesperidin alone or in combination with diosmin, as described above [95,97]. Moreover, when administered subcutaneously to CD-1 mice, hesperidin inhibited TPA-induced tumor promotion, although it did not inhibit DMBA-induced tumor initiation [164]. Later, they documented the protective effect of hesperidin against the TPA-stimulated infiltration of neutrophils, suggesting its potential as a chemopreventive agent against tumor promoter-induced inflammation and hyperplasia [165]. Daily administration of hesperetin (20 mg/kg BW) *per os* for 15 weeks inhibited rat colon carcinogenesis during and after DMH initiation [166]. Further, in rats with DMBA-induced mammary gland tumors, pretreatment with hesperetin (50 mg/kg BW/day) significantly reduced the tumor burden and the overexpression of the proliferating cell nuclear antigen (PCNA), as well as restoring the decreased Bcl-2 and increased Bax expression. By contrast, in the liver of mice treated with DMBA, at a dosage of 10 mg/kg BW, it prevented DNA fragmentation and decreased Bax expression and cleaved caspase-3, caspase-9 and PARP [167]. This study suggests that hesperetin may act as either pro-apoptotic or anti-apoptotic agent depending on the circumstance [167]. Attenuation of BP-induced lung cancer afforded by hesperidin supplementation (25 mg/kg BW) has also been reported [168]. Finally, dietary administration of hesperetin at 1000 ppm and 5000 ppm significantly deterred xenograft growth in athymic mice ovariectomized and transplanted with aromatase-overexpressing MCF-7 cells, while no such effect was observed in mice treated with apigenin or naringenin. Western blot analysis indicated that cyclin D1, CDK4, and Bcl-XL were reduced in the tumors of hesperetin-treated mice, and there are also results suggesting that the flavonone reduces plasma estrogen [169].

Didymin and poncirin are two flavanones that have been investigated less. However, studies have shown their ability to induce the extrinsic apoptosis pathway in human non-small cell lung cancer cells [170] and gastric cancer cells [171], respectively. 

Anthocyanidins and anthocyanins occur ubiquitously in the plant kingdom and confer the bright red, blue, and purple colors to fruits and vegetables. In CF, they are found most commonly in oranges, predominantly as mixture of them. Several investigations have shown the antiproliferative effects of anthocyanidins and anthocyanins both in vitro (towards multiple cancer cell types) and in vivo [172]. The main characteristics of the studies presented in this section are reported in Table 3.

### 3.3. Inhibition of Tumor Progression: Focus on Angiogenesis and Metastatization

Both development and progression of solid neoplasms requires rapid and persistent growth of new blood vessels (neo-angiogenesis) around the cancer tissue to supply the growing tumor with nutrients and oxygen. Cancer cells can stimulate angiogenesis by secreting angiogenesis-promoting growth factors, such as the vascular endothelial growth factor (VEGF), the most important endothelial cell-selective mitogen in vitro. VEGF also produces a substantial increase in vascular permeability that allows tumor cells access to the bloodstream, thereby linking angiogenesis and metastases with a poor prognosis [91].

It has been reported that some flavonoids, including naringin, apigenin, and rutin, are able to inhibit VEGF release in MDA human breast cancer cells [173], and VEGF and transforming growth factor-β1 (TGF-β1) in the GL-15 glioblastoma cell lines [174]. Several findings suggest that apigenin can be considered a natural anti-angiogenic compound. Indeed, it reduces VEGF transcriptional activation via hypoxia-inducible factor 1 (HIF-1) pathway in A549 lung cancer cells, and inhibits angiogenesis in the tumor tissues of nude mice [175]. The inhibition of HIF-1 and VEGF expression has been described in different cancer cells in normoxic or hypoxic conditions [176]. The Authors described the inhibition of tumor angiogenesis using both chicken chorioallantoic membrane and Matrigel plug assays [176]. Apigenin-induced reduction of neo-angiogenesis in the human umbilical vein endothelial cell (HUVEC) seems to be mediated by inhibition of matrix-degrading proteases [177]. Recently, it has been shown that apigenin may act by modulating the inflammatory cytokine IL-6/activators of transcription 3 (STAT3) (IL-6/STAT3) signaling pathways in HUVEC cells. Angiogenesis inhibition resulted in modulation of the activation of extracellular signal-regulated kinase-1/2 (ERK 1/2) signaling triggered by IL-6, as well as in a marked reduction in the proliferation, migration, and morphogenic differentiation of endothelial cells. These effects were coupled with reduced expression of the IL-6 signal transducing receptor-alpha (IL-6Rα) and suppression of cytokine signaling (SOCS3) protein, as well as the secretion of extracellular matrix metalloproteinase (MMP)-2 [178].

Other *Citrus* flavonoids have been evaluated for their potential anti-angiogenic capability. Lam et al. [179] demonstrated the anti-angiogenic activity of some polymethoxylated flavonoids, including hesperetin and nobiletin, both in vitro (HUVEC cells) and in vivo (the zebrafish embryo model). The structure–activity relationship (SAR) analysis indicated that a flavonoid with a methoxylated group at the C3′ position offers stronger anti-angiogenic activity, whereas the absence of a methoxylated group at the C8 position causes lower lethal toxicity in addition to enhancing anti-angiogenic activity. Anti-angiogenic activity of nobiletin in vitro and in vivo previously reported by Kunimasa et al. [180], gave an in-depth description of the mechanisms underlying its inhibitory action on multiple functions of the proliferation, migration, and tube formation of HUVEC cells. Wang et al. [181] reported nobiletin to inhibit tumor growth and angiogenesis by reducing VEGF expression of K562 cells xenograft in nude mice. Moreover, quercetin inhibited tube formation in HUVEC cells and suppressed the angiogenic process in a chick chorioallantoic membrane assay [182]. Interestingly, the flavonoid quercetin possessed strong inhibitory effects on vessel formation and on endothelial cell proliferation, and concomitantly showed strong antioxidant activity [183].

Many studies have reported that flavonoids, many of which are abundant in the *Citrus* genus, are an effective natural inhibitor of cancer invasion and metastasis [184]. In particular, tangeretin and nobiletin appear to be able to inhibit the progression phase of carcinogenesis.

In MCF-7/6 breast cancer cells, tangeretin was found to upregulate the function of the E-cadherin/catenin complex, which consequently led to firm cell–cell adhesions and inhibited cell invasion [185]. In brain tumor cells, nobiletin, and to a lesser extent, tangeretin, exhibited inhibitory activity on the adhesion, migration, invasion, and secretion of MMP-2/MMP-9. In glioblastoma, nobiletin inhibited human U87 and Hs683 glioma cell growth and migration by arresting cell cycle and suppressing the MAPK and Akt pathways [69]. Naringin inhibited the invasion and migration of glioblastoma U87 MG cells by increasing the expression of tissue inhibitors of metalloproteinases (TIMP-1 and TIMP-2), thereby decreasing the expression and proteinase activity of MMP-2 and MMP-9 and enhancing the focal adhesion kinase (FAK)/MMPs pathway [138]. Moreover, naringin inhibited cell migration and invasion of chondrosarcoma cells via vascular cell adhesion molecule 1 (VCAM-1) down-regulation by increasing miR-126 [186], while in bladder cancer cells it downregulated the Akt and MMP-2 pathways [187]. In an experimental model of pulmonary metastasis generated by inoculating albino Swiss mice with highly metastatic murine melanoma cells B16F10, diosmin reduced the number of metastatic nodules in the lung more effectively than tangeretin and rutin [188]. Furthermore, oral administration of naringenin or hesperitin reduced the number of lung metastases in C57BL6/N mice inoculated with B16F10 cells, and increased survival time after tumor cell inoculation [189]. In addition, in a breast cancer resection model that mimics clinical situations after surgery, orally administered naringenin significantly decreased the number of metastatic tumor cells in the lung and extended the life span of tumor resected mice. Both in vitro and in vivo experimental results have further demonstrated that relief of immunosuppression caused by regulatory T cells might be the fundamental mechanism underlying metastasis inhibition by naringenin [190]. Some reports have illustrated the mechanisms by which nobiletin may reduce tumor invasion and metastasis in vitro. In human fibrosarcoma HT-1080 cells stimulated with TPA, it directly inhibited the phosphorylation of mitogen-activated protein/extracellular signal-regulated kinase (MEK), thereby suppressing either the sequential phosphorylation of extracellular regulated kinases (ERK) and the expression of MMP [191]. MMP-1 and -9 expression were suppressed by nobiletin in fibrosarcoma cells with an associated increase in tissue inhibitors of MMPs [192]. Additionally, MMP-7 was down-regulated in colorectal cells [193], while MMP-2 in human nasopharyngeal carcinoma cells [194]. Nobiletin exerts antimetastatic effects on human breast cancer cells [195] through the down-regulation of both CXC chemokine receptor type 4 (CXCR4) and MMP-9 via a mechanism involving NF-κB inhibition and MAPKs activation. Minagawa et al. [196] showed that pro-MMP-9 activity was inhibited by nobiletin in gastric cell lines, and reported a significant reduction in the peritoneal dissemination of stomach cancer nodules when the polymethoxylated flavone was administered subcutaneously to severe combined immune deficient (SCID) mice. Moreover, nobiletin has been shown to reduce adhesion, invasion, and migration of highly metastatic human gastric adenocarcinoma AGS cells by inhibiting the activation of FAK and PI3K/Akt signals, which in turn downregulates MMP-2 and -9 expression and activity [197]. Finally, nobiletin inhibited the epithelial–mesenchymal transition of human non-small cell lung cancer cells by antagonizing the TGF-β1/Smad3 signaling pathway, thus prohibiting the growth of metastatic nodules in the lungs of nude mice [198].

Treatment of MDA-MB-231 breast tumor cells with apigenin (ranging from 2.5 to 10 μg/mL) led to a partial decrease in urokinase-plasminogen activator (uPA) expression and completely inhibited phorbol 12-myristate 13-acetate (PMA)-induced MMP-9 secretion [199]. Apigenin also inhibited hepatocyte growth factor (HGF)-induced migration and invasion and decreased HGF-stimulated integrin β4 and Akt phosphorylation in MDA-MB-231 cells. It also inhibited HGF-promoted metastasis in nude mice and in chick embryos [200]. In prostate cancer, the motility and invasion of PC3-M cells were inhibited by apigenin through a FAK/Src signaling mechanism [201]. In ovarian cancer, it inhibited FAK-mediated migration and invasion of A2780 cells, and repressed spontaneous metastasis formation on the ovaries of nude mice following inoculation with A2780 cells [202]. In cervical cancer, apigenin inhibited the motility and invasiveness of HeLa cells [203]. Moreover, its administration significantly decreased the incidence of cancer metastasis in AOM-induced intestinal adenocarcinoma in rats [204]. Noh et al. [205] further reported that this flavone inhibited PMA-induced migration and invasion of human cervical carcinoma Caski cell line via the suppression of p38 MAPK-dependent MMP-9 expression. Finally, intraperitoneal administration of apigenin and quercetin into syngeneic mice injected with B16-BL6 melanoma cells resulted in a significant delay in tumor growth and lungs metastases, with flavonoids being more effective than tamoxifen [206].

Over the last decade, there has been extensive researches into the potential anti-invasive role of quercetin. In breast cancer, the invasive activity of PMA-induced MCF-7 cells was blocked by the flavonol by reducing MMP-9 expression and by blocking activation of the protein kinase C (PKC)/ERK/AP-1 signaling cascade [207]. In MDA-MB-231 cells the anti-invasive effect was mediated by inhibiting MMP-3 activity [208]. In PC-3 prostate cancer cells, quercetin (50 and 100 μM for 24 h) decreased MMP-2/MMP-9 expression [209] and downregulated the mRNA of uPA, uPA receptor (uPA-R), EGF, and EGF receptor (EGF-R), thereby inhibiting invasion and migration [210]. In human glioblastoma U87 cells, quercetin blocked PMA-induced migration and invasion by inhibiting ERK-dependent COX-2 activation and MMP-9 activity [211], while in the DAOY medulloblastoma cell line, it reduced both Met-induced cell migration and HGF-mediated Akt activation [212]. Moreover, quercetin decreased the invasiveness of A431 epidermal cancer cells by increasing EGF-depressed E-cadherin, by down-regulating both epithelial–mesenchymal transition (EMT) markers and MMP-9, leading to the restoration of cell–cell junctions [213]. In addition, it inhibited cell–matrix adhesion, migration, and invasion of HeLa cells [214] and inhibited the motility and invasion of murine melanoma B16-BL6 cells by decreasing pro-MMP-9 via the PKC pathway [215]. The administration of quercetin to DMBA-induced mammary carcinoma rats has been reported to significantly decrease both tissue type plasminogen activator (t-PA) and u-PA [216]. Lastly, didymin was observed to suppress phthalate-mediated breast cancer cell proliferation, migration, and invasion, suggesting that it is capable of preventing phthalate ester-associated cancer aggravation [217]. Table 4 summarizes the essential features of the studies on the anti-angiogenic and anti-metastatic activity of *Citrus* flavonoids.

## 4. Anti-Cancer Properties of *Citrus* Juices and Extracts

As described above, a number of studies have investigated the anti-cancer effect of single *Citrus* flavonoids as pure compounds. However, few studies have focused on the biological activity of *Citrus* juices and extracts. A very interesting paper [218] explains why a single bioactive compound may not replicate the same effect as the phytocomplex in which it is contained. Indeed, often, even at high concentrations, no single active principle can replace the combination of natural phytochemicals present in an extract in achieving the same magnitude of pharmacological effect. Liu [218] suggests that the additive and synergistic effects of phytochemicals in fruits and vegetables are responsible for these potent antioxidant and anti-cancer activities, and that the benefits of a diet rich in fruits and vegetables is attributable to the complex mixture of phytochemicals present in whole foods. This concept has been supported through data obtained employing several nutraceuticals by Surh [10].

In line with this, some preclinical studies have indicated that *Citrus* juices and extracts may reduce cancer formation and progression. To the best of our knowledge, So et al. [219] were the first to show that concentrated *Citrus sinensis* (orange) juice inhibits the development of mammary tumors induced by 5 mg of DMBA in rats, also suggesting the anti-cancer properties of naringin and quercetin. Two years later, the same Authors [220] showed that a double-strength orange juice administration inhibited DMBA-induced mammary tumorigenesis in rats more effectively than double-strength grapefruit juice. Moreover, Miyagi and coworkers [221] showed that orange juice inhibits AOM-induced colon cancer in male rats, suggesting that flavonoids and limonoid glucosides might be responsible for this anti-cancer activity. *Citrus reticulata* (mandarin) juice has also long been investigated regarding its antitumoral activity. In particular, studies have demonstrated the capability of mandarin juice to suppress the chemically-induced carcinogenesis in colon, tongue, and lung cancers, especially when it is supplemented with added amounts of flavonoids, such as beta-cryptoxanthin and hesperidin [222,223,224,225]. Recently, we have investigated the effects of a flavonoid-rich extract from mandarin juice (MJe) on three human anaplastic thyroid carcinoma cell lines (CAL-62, C-643, and 8505C cells), showing that MJe reduced cell proliferation through a block of the cell cycle in the G2/M phase, accompanied by low cell death due to autophagy. Moreover, MJe reduced activity of MMP-2, thus decreasing cell migration [226]. In another study, Vanamala and coworkers [139] showed that grapefruit juice and limonin produce suppressive effects on AOM-induced colon carcinogenesis by lowering inducible nitric oxide synthases iNOS and cyclooxygenase-2 COX-2 levels and upregulating apoptosis, thereby reducing the formation of aberrant crypt foci. Furthermore, methanolic extract of lemon fruit triggered apoptosis of MCF-7 human breast cancer cells [227]. An analogous effect was achieved on the same cell line using lemon seed extract [228].

In recent years, *Citrus bergamia* (bergamot) fruit has attracted attention due to its potential anti-cancer effects. In particular, we have shown that bergamot juice (BJ) to reduce the growth rate of different cancer cell lines by different molecular mechanisms, depending on cancer type. In SH-SY5Y human neuroblastoma cells, BJ stimulated the cell cycle arrest in the G1 phase without inducing apoptosis, and caused a modification in cellular morphology associated with a marked increase in detached cells. The inhibition of adhesive ability onto different physiologic substrates and onto endothelial cell monolayer was correlated with BJ-induced impairment of actin filaments and with the reduction in the expression of the active form of FAK, in turn causing inhibition of cell migration [229]. Contrariwise, in human hepatocellular carcinoma HepG2 cells, we demonstrated that BJ reduces the growth rate through the involvement of p53, p21, and NF-κB pathways, as well as the activation of both intrinsic and extrinsic apoptotic pathways [230]. Moreover, we documented that the BJ-induced reduction of both cell adhesiveness and motility could be responsible for the slight inhibitory effects on lung metastasis colonization observed in an animal model of spontaneous neuroblastoma metastasis formation in SCID mouse [231]. In order to assess which bioactive component of BJ was responsible for its antitumor activity, we focused on the flavonoid-rich fraction from bergamot juice (BJe). Our results suggested that BJe inhibits HT-29 human colorectal carcinoma cell growth and induces apoptosis through multiple mechanisms. Molecular assays revealed that higher concentrations of BJe increase ROS production, which causes a loss of mitochondrial membrane potential and oxidative DNA damage. Lower concentrations of BJe inhibited MAPK pathways and modified apoptosis-related proteins, which in turn induced cell cycle arrest and apoptosis [232].

It is well known that chronic inflammation might lead to carcinogenesis, and that both inflammatory cells and cytokines contribute to tumor growth, progression, and immunosuppression [233]. Moreover, there is evidence to support the hypothesis that dysregulation of both inflammatory and redox pathways in tumor cells and in their stromal environment play an essential role in tumorigenesis, invasion, and systemic spread [234]. Furthermore, inflammatory pathways are constitutively active in most cancers. Therefore, the use of medicines with antioxidant and anti-inflammatory activities is desirable in oncological applications. In addition, although natural remedies are not risk free, they are generally safer than both synthetic and biological drugs. In this context, we have recently shown that BJe has antioxidant properties [235,236] and is able to suppress pro-inflammatory responses in both in vitro [237,238] and in vivo models [239,240]. Interestingly, evidence showing that BJ did not significantly affect the viability of normal human diploid fibroblast WI-38 cells [229], as well as not provoking any apparent sign of systemic toxicity [231], together with its antimicrobial activity [241,242] and favorable safety/efficacy balance [243], reveals the potential of BJe as an anti-cancer remedy, highlighting that it could represent a novel strategic approach in oncology field. 

Other studies have been performed using extracts of *Citrus* derivatives. For examples, Mak and collaborators [244] reported that an extract from the pericarpium of *Citrus reticulata* inhibited the proliferation of murine myeloid leukemia WEHI 3B cells and induced their differentiation into macrophages and granulocytes, identifying nobiletin and tangeretin as the active components. Kim and coworkers [245] reported the anti-proliferative and pro-apoptotic effects of a *Citrus reticulata* Blanco peel extract on the human gastric cancer cell line SNU-668. Park et al. [246] used a flavonoid extract from the peel of Korean *Citrus aurantium* L. and found it was able to induce cell cycle arrest and apoptosis in A549 lung cancer cells, while Han and collaborators [247] suggested that a crude methanol extract of *Citrus aurantium* L. peel should induce caspase-dependent apoptosis through the inhibition of Akt in U937 human leukemia cells. Two animal studies using an orange peel extract abundant in polymethoxyflavones, showed its ability to reduce the development of hyperplastic lesions and to increase apoptosis in ductal epithelial cells of mouse mammary glands [55], and to inhibit intestinal tumorigenesis in Apc^(Min/+)^ mice [54]. Moreover, the ethanolic extract of peel from *Citrus aurantifolia* increased the sensitivity of MCF-7 cells to doxorubicin, enhancing both cell cycle arrest and apoptosis [248]. Similarly, total flavonoids from *Citrus paradisi* Macfadyen peel, when combined with arsenic trioxide, produced a synergistic effect in reducing the proliferation of leukemia cells and triggering apoptosis [249], suggesting that *Citrus* extracts could be used as co-adjuvants in cancer therapy. Finally, we have shown that the bergamot essential oil (BEO) obtained by rasping the peel of *Citrus bergamia* fruits decreased the growth rate of SH-SY5Y neuroblastoma cells [250] by a mechanism correlated to both apoptotic and necrotic cell death [251]. Table 5 summarizes the main characteristics of the above investigations into the anti-cancer properties of *Citrus* juices and extracts.

## 5. Epidemiological Studies

Over the last few decades, epidemiological and clinical studies have suggested that regular intake of CF may protect against cancer development. The majority of the clinical evidence supporting the potential anti-cancer effects of *Citrus* is derived from case–control studies. One of the first population-based case-control studies evaluating whether *Citrus* intake is associated with a reduced cancer risk was carried out in Shanghai at the end of the 1990s. The aim of this study was to investigate the association between dietary factors and risk of nasopharyngeal carcinoma (NPC), Yuan et al. [252] found that high intake of oranges and tangerines was associated with a statistically significant reduction in the risk of NPC. The study included 935 NPC patients aged 15 to 74 years interviewed by a questionnaire. Authors concluded that oranges and tangerines are a rich source of vitamin C that can block nitrosamine formation, thereby offering a biological rationale for the anti-NPC effect. In the 1990s, Bosetti et al. [253] conducted a hospital-based case–control study in three areas of northern Italy on 304 patients affected by a squamous cell carcinoma of the esophagus and 743 controls who were asked to complete a questionnaire. The results of this observational study provide further evidence to support the theory that consumption of CF is inversely related to esophageal cancer risk. Steevens et al. [254] reached the same conclusions when studying a Netherlands cohort. High intake of CF has also been associated with reduced risk of cancer of the oral cavity and pharynx [255]. Some years later, the same research group, performed a population-based case control study recruiting subjects in Northern Italy and Swiss Canton of Vaud in the 1990s showed that intake of CF may also reduce laryngeal cancer [256]. In line with these findings, a prospective study on 42,311 US men in the Health Professionals Follow-up Study [257] reported that histologically-diagnosed oral premalignant lesions were suppressed by consumption of CF and CF juices (30% to 40% lower risk), thus upholding results previously obtained in Europe on smaller subject groups. Interestingly, a meta-analysis showed that the CF consumption exerts the strongest protective effect against oral cancer compared to all other kinds of fruits [258]. Pourfarzi et al. [259] reported that regular intake of fruits could reduce the risk of gastric cancer by more than half. In particular, consumption of CF was more protective than all other fruits, and subjects eating them more than three times per week had about a 70% lower risk than those who never or infrequently ate CF. The beneficial effects of CF with respect to stomach cancer prevention were confirmed by a more recent cohort study performed in Netherlands [254]. Epidemiological data from a network of case–control studies strengthen the hypothesis that increasing consumption of CF may reduce the risk of cancers of the digestive and upper respiratory tract [260]. Gonzalez and co-workers [261] also observed a significant inverse correlation between total CF ingestion and gastric cancer risk.

However, the possibility that intake of CF can prevent the development of colon cancer is quite controversial [262,263]. A large population-based case–control study was conducted on Chinese women in Shanghai by interview. Tangerines, oranges, and grapefruits were found to be inversely associated with breast cancer risk among pre-menopausal women, but the same data was not found to be statistically significant in post-menopausal women [264]. However, a more recent study revealed a significant protective effect against breast cancer by oranges, orange juice, and other CF [265]. Intake of either CF [266] or orange, grapefruit, and their juice [267] also reduced the risk of developing pancreatic cancer. Moreover, CF intake also seems to be inversely associated with prostate cancer risk [268], and high consumption of both tangerines and oranges was found to be protective against melanoma [269]. Recently, a prospective study showed that *Citrus* consumption, especially if eaten daily, was correlated with reduced incidence of all cancers, although significant results were only obtained for prostate and pancreatic cancer [270]. About 40,000 Japanese patients of Ohsaki were followed for up to 9 years to assess the *Citrus* consumption by a self-administered questionnaire. This study overcomes the bias of other studies described above due to their retrospective nature, confirming the ability of CF to reduce risk of first and second primary tumors [270]. Interestingly, one prospective study indicated that high intake of CF may confer protection against the development of second primary cancers, particularly in the lung [271].

Furthermore, meta-analyses have confirmed the relationship between CF intake and decreased risk of cancers. In particular, Bae et al. [272] have provided evidence for the protective effects of high CF ingestion against stomach cancer risk. Another quantitative systematic review [273] has reported an inverse association between CF consumption and pancreatic cancer risk, although the effect was limited due to the weakness of study design. More recently, different meta-analyses have highlighted an inverse association between CF intake and the risk of various types of cancers, such as breast cancer [274], bladder cancers [275,276,277], and esophageal cancer [278]. A very recent systematic literature review of prospective studies on CF intake and risk of esophageal and gastric cancers revealed only a marginally significant decreased risk of esophageal cancer and reported no significant inverse association for gastric cardia cancer, but data are still limited [279].

However, some researchers have reported the ineffectiveness of CF in cancer prevention. For instance, the results from a large European prospective cohort suggested that higher consumption of fruits and vegetables is not associated with decreased risk of pancreatic cancer [280]. Moreover, Bae and coworkers [273] found no association between CF intake and risk of prostate cancer.

The reasons for this variability are multi-factorial, but probably reflect the ability of *Citrus* flavonoids to interact with their molecular targets, and are due to their poor bioavailability and issues linked to the study design. The latter include: fluctuations in CF intake, the qualitative/quantitative composition of CF, the relative concentration of bioactive molecules, the eventual standardization (in the case of natural remedies), the patient’s compliance with the instructions provided by the investigator, and other numerous possible confounding elements. Nevertheless, although evidence linking CF intake and cancer prevention are conflicting, epidemiological data seem to support the hypothesis of some protection against certain types of cancer by CF. Table 6 collects the studies presented in this paragraph.

## 6. Concluding Remarks

Overall, knowledge about the effects of flavonoids on cancer development has progressively grown over recent years, as well as people’s desire to maintain good health through increasing use of nutraceuticals, functional foods, and natural remedies. Numerous in vitro and in vivo studies have shown the ability of flavonoids to exert anti-cancer effect, and some epidemiological studies support this hypothesis. Moreover, evidence showing that flavonoids act not only as free radical scavengers but also as modulators of several key molecular events implicated in cell survival and death, has heightened scientific interest in these plant secondary metabolites. The main sources of dietary flavonoids for humans are fruits, especially *Citrus* fruits and their juices, along with vegetables, wine, and tea. Over the last few decades, experimental research and epidemiological studies indicate that CF and their flavonoids could have anti-tumor properties. The experimental results discussed in this review have clearly shown that *Citrus* flavonoids may act as chemopreventive and chemotherapeutic agents, either as single agents or as co-adjuvants for other drugs. However, the majority of studies on the anti-cancer potential of *Citrus* extracts and their single components have been carried out in in vitro and in vivo models, and the extrapolation of preclinical results for human use is difficult to achieve, particularly, but not solely, due to problems linked to pharmacokinetics. Indeed, the modest bioavailability of flavonoids and their limited duration of action are the main obstacles restricting their clinical use. Some flavonoids, such as quercetin and anthocyanins, can be absorbed at the gastric level, while others—resistant to acid hydrolysis in the stomach— intact reach the intestine where are absorbed. However, most of the flavonoids present in food are esters, glycosides, or polymers, which are not absorbed in their native form because of their extensive modification by intestinal enzymes such as β-glucosidases and lactase-phlorizin hydrolase present in the resident bacterial flora. Moreover, flavonoids may be subjected to intestinal and hepatic first-pass extraction that can further affect their bioavailability. However, some metabolic reactions lead to the formation of biologically active metabolites. While some flavonoids undergo an extensive pre-systemic elimination, others are less vulnerable, depending on their chemical structure. Inter-individual variations have also been observed, probably due to the different composition of the colonic microflora which can affect their metabolism in different ways. Nevertheless, despite bioavailability problems, numerous experimental and clinical data have demonstrated the ability of *Citrus* flavonoids to exert important systemic pharmacological effects [14,281,282]. In addition, *Citrus* flavonoids also display neuroprotective effects [283,284], suggesting that they are able to cross the blood–brain barrier. One explanation for the apparent discrepancy between the poor bioavailability of flavonoids and their biological activity in humans would be to assume that a significant part of the biological actions exhibited by *Citrus* flavonoids are due to their active metabolites. Another hypothesis is the underestimation of plasma concentration and half-life due to their large volume of distribution values, to their relatively rapid post-systemic metabolization, and to the limits of assay sensitivity. In addition, to the best of our knowledge, there are few appropriately designed clinical trials to assess both pharmacological efficacy and pharmacokinetic profile of the bioactive molecules contained in CF. However, clinical studies evaluating the effectiveness of CF extracts or flavonoids mixtures in which one or more was from CF are a little more numerous. This evidence, together with the findings of other Authors [10,218,285], strengthens our thesis that given the multi-factorial pathogenesis of cancer, the complex mixture of phytochemicals present in a whole extract acts better than a single constituent. This is because all molecules present in a phytocomplex can simultaneously modulate different targets of action in both human cells and microorganisms, leading to a pool of pharmacological effects contributing together to improve the patient’s health. On the bases of several preclinical and epidemiological studies summarized in this review, we believe that regular intake of CF and their derivatives, linked to a healthy life style, might be an important way to reduce cancer risk.

## Figures and Tables

**Table 1:** Main mechanisms through which *Citrus* flavonoids may act as anti-cancer drugs.

**Mechanism by Which Citrus Flavonoids May Fight against Cancer**
Antioxidant activity, thus counteract oxidative stress
Anti-inflammatory effect
Phase II enzyme induction, hence enhancing detoxification
Phase I enzyme inhibition, thus stopping activation of carcinogens
Inhibition of cell proliferation
Inhibition of oncogene and/or induction of tumor suppressor gene
Induction of cell-cycle arrest
Induction of apoptosis
Inhibition of signal transduction pathways
Anti-angiogenic effect
Inhibition of cell adhesion, migration and invasion

**Table 2:** Studies investigating the ability of *Citrus* flavonoids to inhibit the initiation phase of carcinogenesis.

Initiation Phase			
Flavonoid	Concentration/Dose	Experimental Model	Reference
Quercetin	0.1–5.0 μM	HgCl_2_/MeHg-treated HepG2 cells	[26]
Naringenin	10–80 μM	Ferrous sulfate-exposed LNCaP cells	[27]
Naringenin	200 mg/kg	NDEA-treated rats	[28]
Naringenin	200 mg/kg	NDEA-treated rats	[29]
Naringin	50–500 mg/kg	Ifos-treated mice	[30]
Naringin	50–500 mg/kg	Dau-treated mice	[31]
Naringin	10–200 mg/kg	DMH-injected rats	[32]
Hesperidin	50–400 mg/kg	Cyclophosphamide-treated mice	[33]
Naringin, apigenin, hesperetin	300 μg/plate	Aflatoxin B1-exposed *Salmonella typhimurium* TA100	[34]
Diosmin, naringenin, naringin, rutin	0.25–1.0 μM	Heterocyclic amines-exposed *Salmonella typhimurium* TA98	[37]
Apigenin	10–100 μM	308 and HCT116 cells	[38]
Apigenin	2.5 and 5 mg/kg	BP-treated mice	[39]
Quercetin, kaempferol, myricetin, apigenin	5–25 μM	COS-1 cells	[40]
Apigenin	1–50 μM	DMBA/TPA-exposed mice	[41]
Apigenin	5 and 10 μmoles in 200 μL	UV-A/B-exposed SKH-1 mice	[42]
Apigenin, naringenin	0.1% and 0.02%	AOM-treated rats	[43]
Hesperidin	30 mg/kg	DMBA-treated rats	[44]
Hesperetin	20 mg/kg	DMH-treated rats	[45]
Tangeretin	50 mg/kg	DMBA-treated rats	[46]
Nobiletin	160 and 320 nM	DMBA/TPA-exposed mice	[47]

AOM: azoxymethane; BP: benzo(α)pyrene; Dau: daunorubicin; DMBA: 7,12-dimethylbenz(α)anthracene; DMH: 1,2-dimethylhydrazine; Ifos: ifosfamide; NDEA: *N*-diethylnitrosamine; TPA: tetradecanoyl-13-phorbol acetate.

**Table 3:** Studies on the ability of *Citrus* flavonoids to inhibit tumor development.

Promotion Phase			
Flavonoid	Concentration/Dose	Experimental Model	Reference
Quercetin, taxifolin, nobiletin, tangeretin	2–8 μg/mL	HTB43 cells	[48]
Tangeretin	50–100 μM	HL-60 cells	[49]
Tangeretin	2.7–27 μM	HL-60 cells	[50]
Tangeretin, nobiletin	54 μM (tangeretin)	MDA-MB-435, MCF-7, and HT-29 cells	[51]
	100–200 μM for MDA-MB-435		
	60 μM for MCF-7		
	200 μM for HT-29 (nobiletin)		
Tangeretin	10–50 μM	COLO 205 cells	[52]
Nobiletin	20–200 μM	TMK-1, MKN-45, MKN-74, and KATO-III cells	[53]
Tangeretin	10^−7^–10^−4^ M	T47D cells	[56]
Nobiletin	20–30 μM	H_2_O_2_-treated SH-SY5Y cells	[57]
Tangeretin, nobiletin	IC_50_ 4 mg/mL	Brain tumor cells	[58]
Tangeretin	150 μM	A2780/CP70 and 2008/C13 cells	[59]
Tangeretin	5–240 μM	AGS cells	[60]
Nobiletin	1 × 10^−7^–5 × 10^−4^ mol/L	TRAP rats	[61]
Nobiletin	0.05%	PhIP-treated rats	[62]
Nobiletin	0.01%–0.05%	AOM-treated rats	[63]
Chrysin, quercetin, nobiletin	100 ppm	AOM-treated mice	[64]
Nobiletin	100 ppm	AOM/DSS-treated mice	[65]
Nobiletin	1.25–80 μM	A549 cells	[66]
Nobiletin	10^−3^ M	MH1C1 and HepG2 cells	[67]
Nobiletin	10–100 μM	C6 cells	[68]
Nobiletin	20–100 μM	U87 and Hs683 cells	[69]
Nobiletin	0–200 μM	AGS, MKN-45, SNU-1, and SNU-16 cells	[70]
Nobiletin	0–160 μM	HL-60, U937, THP-1, OCI-AML3, and MV4-11 cells	[71]
Nobiletin	0.05 wt%	AOM/DSS-treated CD-1 mice	[72]
Apigenin	1–100 μM	MDA-MB-453 cells	[73]
Apigenin	0–40 μM	MCF-7, MCF-7 HER2, SK-BR-3 cells	[74]
Apigenin	10–70 μM	MDA-MB-453, BT-474, SKBr-3, MCF-7, and HBL-100 cells	[75]
Apigenin	0–60 μM	HT-29 and MG63 cells	[77]
Apigenin	10–50 μM	HDF cells	[78]
Apigenin	IC_50_: 7.8 μg/mL for MCF-7 and 8.9 μg/mL for MDA-MB-468 cells	MCF-7 and MDA-MB-468 cells	[80]
Apigenin	1–100 μM	BxPC-3 and MiaPaCa-2 cells	[81]
Apigenin	6.25–100 μM	AsPC-1, CD18, MIA PaCa2, and S2-013 cells	[82]
Apigenin	10–100 μM	BxPC-3 and PANC-1 cells	[83]
Apigenin	10–80 μM	LNCaP cells	[84]
Apigenin	1–20 μM	DU145 cells	[85]
Apigenin	0–80 μM	SW480, HT-29, and Caco-2 cells	[86]
Apigenin	10–10 μM	HCT-116, SW480, HT-29, and LoVo cells	[87]
Apigenin	20–50 μg/mouse	22Rv1 and PC-3 cells-implanted mice	[88]
Apigenin	50 μM	SH-SY5Y cells	[89]
Apigenin	15–60 μM and 25 mg/kg	NUB-7, LAN-5, and SK-*N*-BE cells and NUB-7 inoculated xenograft mice	[90]
Flavonids	25–250 μM	HT-29, Caco-2, LLC-PK1, and MCF-7 cells	[92]
Diosmin	0–120 μM and 15 mg/kg	HA22T cells and HA22T xenograft mice	[93]
Diosmin	50–250 μM	DU145 cells	[94]
Diosmin, hesperidin	1000 ppm	MNAN-injected rats	[95]
Diosmin, hesperidin	1000 ppm	4-NQO-exposed rats	[96]
Diosmin, hesperidin	500–1000 ppm	OH-BBN-exposed rats	[97]
Diosmin, hesperidin	1000 ppm	AOM-injected rats	[98]
22 flavonoids	0–10 μM	HL-60, A431, SK-OV-3, HeLa, HOS cells	[99]
Quercetin	0–100 μM	Caco-2 and HT-29 and IEC-6 cells	[102]
Quercetin	0–50 μM	Prostate and skin cells	[104]
Quercetin	0–50 μM	MDA-MB-231, MDA-MB-453, AU565, BT483, BT474, and MCF-7 cells	[105]
Quercetin	0–10 μM	SK-Br-3 and SK-Br-3-Lap R cells	[106]
Quercetin	2.5–40 μM	MDA-MB-231, MCF-7, and MCF-10A cells	[107]
Quercetin	1–10 μM	MCF-7ADR-resistant cells	[108]
Naringenin	0–1 mM	HL-60 cells	[110]
Naringenin	0.02–2.85 mmol	HT-29 cells	[112]
Naringenin	10 μM	MCF-7 cells	[113]
Naringenin	0–400 μM	THP-1 cells	[114]
Naringenin	50–750 μM	HaCaT and A431 cells	[116]
Naringenin	0.1–0.5 mM	HL-60 cells	[117]
Naringenin	100 μM	A549, H460, and WI-38 cells	[118]
Naringenin, hesperetin, apigenin	50 μM	MCF-7 and NCI-H460 cells	[119]
Naringenin, kaempferol	25–100 μM	HK-2 cells	[121]
Naringenin	10 mg/kg	Rats	[122]
Naringenin, naringin	0.7 mg/kg (naringenin) and 2.4–9.4 mg/kg (naringin)	Rats	[123]
Naringenin	100 μM	A549, MCF-7, HepG2, and MCF-7/DOX cells	[124]
Naringin, naringenin, quercetin	50 mg/kg (naringin or naringenin) and 100 mg/kg (quercetin)	Rats	[125]
Naringenin	200 mg/kg	MNNG-treated rats	[126]
Naringenin	200 mg/kg	MNNG-treated rats	[127]
Naringenin	50 mg/kg	C6 cells-injected rats	[128]
Naringin, naringenin	2.5%	Hamsters	[129]
Naringin	250–2000 μM	SiHa cells	[130]
Naringin	1000 μmol/L	HeLa cells	[131]
Naringin	0–3200 μM	HeLa and A549 cells	[132]
Naringin	50–200 μM and 100 mg/kg	MDA-MB-231, MDA-MB-468, and BT-549 cells/MDA-MB-231 xenograft mice	[133]
Naringin	0–150 μM	5637 and T24 cells	[134]
Naringin	1.2–3 mM	AGS cells	[137]
Naringin	50–200 μM	MDA-MB-231, MDA-MB-468, and BT-549 cells	[138]
Naringin	200 mg/kg	AOM-injected rats	[139]
Naringin	10.25–35 mg/kg	W256 rats	[140]
Naringin	150 mg/kg	Apc^(Min/+)^ mice	[141]
Hesperetin, hesperidin, naringenin, naringin	40–80 μM	HL-60, THP-1, and PMN cells	[143]
Hesperetin	0–200 μM	MCF-7 cells	[144]
Hesperetin	5–100 μM	HT-29 cells	[145]
Hesperetin	0–125 μmol/L	BON cells	[146]
Hesperetin	125–1000 μM	SiHa cells	[147]
Hesperetin	0–600 μM and 10–40 mg/kg	HepG-2, SMMC-7721, and Huh-7/hepatocellular carcinoma xenograft mice	[148]
Hesperetin	20 mg/kg	DMH-injected rats	[149]
Hesperidin, hesperitin, rutin, neohesperidin	25–100 μg/mL	Panc-28 cells	[151]
Hesperidin	1–100 μM	SNU-C4 cells	[152]
Hesperidin	0–200 μM	HepG2 cells	[153]
Hesperidin	0.1–2 mM	HepG2 cells	[154]
Hesperidin	0–100 μM	Ramos cells	[155]
Hesperidin	10–100 μM	NALM-6 cells	[156]
Hesperetin	0–200 μM	MCF-7, MCF-10A, HMEC and MDA-MB-231 cells	[157]
Hesperidin	20–100 μM	MCF-7 cells	[158]
Hesperidin	0–100 μM	HeLa cells	[159]
Hesperidin	0.32–32 μM	Caco-2, CCRF-CEM and CEM/ADR5000 cells	[160]
Hesperetin, quercetin	30 μM	K562, K562/BCRP, MCF7/WT, and MCF7/MR cells	[161]
Hesperidin	0–100 μM	MCF-7, LNCaP, PC-3 and DU-145 cells	[162]
Hesperidin	500 ppm	4-NQO-treated rats	[163]
Hesperidin	1%	DMBA/TPA-treated mice	[164]
Hesperetin	20 mg/kg	DMH-treated rats	[166]
Hesperetin	10–50 mg/kg	DMBA-treated rats	[167]
Hesperidin	25 mg/kg	BP-exposed mice	[168]
Hesperetin	1000–5000 ppm	MCF-7 xenograft mice	[169]
Didymin	0–20 μM	A549 and H460 cells	[170]
Poncirin	50–200 μM	AGS cells	[171]

4-NQO: 4-nitroquinoline 1-oxide; AOM: azoxymethane; DMH: 1,2-dimethylhydrazine; DSS: dextran sulfate sodium; MNAN: *N*-methyl-*N*-amylnitrosamine; MNNG: *N*-methyl-*N*′-nitro-*N*-nitrosoguanidine OH-BBN: *N*-butyl-*N*-(4-hydroxybutyl)nitrosamine; PhIP: 2-amino-1-methyl-6-phenylimidazo[4,5-b]pyridine.

**Table 4:** Studies on the ability of *Citrus* flavonoids to inhibit angiogenesis and metastasis and their characteristics.

Progression Phase			
**Flavonoid**	**Concentration/Dose**	**Experimental Model**	**Reference**
Flavonoids	0.1–100 μmol/L	MDA, U343, and U118 cells	[173]
Rutin	50–100 μM	GL-15 cells	[174]
Apigenin	0–20 μM	A549 cells	[175]
Apigenin	0–30 μM	PC-3, DU145, LNCaP, OVCAR-3, HCT-8, MCF-7 cells	[176]
Apigenin	5 mg/L	HUVEC cells	[177]
Apigenin	25 μM	HUVEC, HMVECs-d-Ad cells	[178]
Hesperetin and nobiletin	0–100 μM and 30 μM	HUVECs cells and zebrafish	[179]
Nobiletin	0–128 μM and 100 μg/egg	HUVEC and HDMEC cells and CAM	[180]
Nobiletin	12.5–50 mg/kg	K562 cells xenograft mice	[181]
Quercetin	0–100 μM and 50–100 nmol/10 μL/egg	HUVEC cells and CAM	[182]
Quercetin	3.13–50 μg/mL	HUVEC cells	[183]
Naringin	0–30 μM	JJ012 and SW1353 cells	[186]
Neringenin	0–300 μM	TSGH-8301 cells	[187]
Tangeretin, rutin, and diosmin	20 mg/animal	B16F10-inoculated mice	[188]
Naringenin and hesperitin	10 μM/20 mg/g of pellets	B16-F10 cells/B16-F10-inoculated C57BL6/N mice	[189]
Naringenin	0–200 μM and 100 mg/kg	4T1 cells/4T1-injected BALB/c and C57BL/6 mice	[190]
Nobiletin	64 μM	TPA-stimulated HT-1080 cells	[191]
Nobiletin	0–64 μM	TPA-stimulated HT-1080 cells	[192]
Nobiletin	0–100 μM	Caco-2, HT-29, Colo205, Colo320DM, LS174T, and LS180 cells	[193]
Nobiletin	0–200 μM	MDA-MB-231 cells	[195]
Nobiletin	0–256 μM/16–64 μM	TMK-1, MKN-45, and St-4 cell/TMK-1-injected mice	[196]
Nobiletin	0–4.5 μM	HepG2, Caco-2, and AGS cells	[197]
Apigenin	2.5–10 μg/mL	MDA-MB231 cells	[199]
Apigenin	0–320 μM	MDA-MB-231, A549, SK-Hep1 cells	[200]
Apigenin	0–50 μM	PC3-M, C4-2B, and DU145 cells	[201]
Apigenin	20/40 μM	A2780 cells	[202]
Apigenin	10–50 μM	HeLa cells	[203]
Apigenin	0.75–1.5 mg/kg	AOM-treated rats	[204]
Apigenin	5–20 μM	PMA-exposed SK-Hep1 and MDA-231 cells	[205]
Apigenin and quercetin	1–10,000 nM/25–50 mg/kg	B16-BL6-injected mice	[206]
Quercetin	80 μM	TPA-treated MCF-7 cells	[207]
Quercetin	0–100 μmol/L	MDA-MB-231 cells	[208]
Quercetin	50–100 μM	PC-3 cells	[209]
Quercetin	25–125 mM	PC-3 cells	[210]
Quercetin	50 μM	TPA-exposed U87 cells	[211]
Quercetin	1–20 μM	HGF-exposed DAOY cells	[212]
Quercetin and luteolin	10–20 μM	A431 cells	[213]
Quercetin	20 to 80 μM/L	HeLa cells	[214]
Quercetin	3.3 × 10^−1^ mM	B16-BL6 cells	[215]
Quercetin	25 mg/kg	DMBA-treated rats	[216]

DMBA: 7,12-dimethylbenz(α)anthracene; HGF: hepatocyte growth factor; TPA: tetradecanoyl-13-phorbol acetate.

**Table 5:** Essential features of the studies evaluating the anti-cancer properties of *Citrus* juices and extracts.

*Citrus* Juices and Extracts	Experimental Model	Reference
*Citrus sinensis* juice	DMBA-injected rats	[219]
*Citrus sinensis* juice	DMBA-injected rats	[220]
*Citrus sinensis* juice	AOM-injected rats	[221]
*Citrus reticulata* juice	AOM-injected rats	[222]
*Citrus reticulata* juice	NNK-injected mice	[223]
*Citrus reticulata* juice	AOM-injected rats	[225]
*Citrus reticulata* juice	CAL-62, C-643, 8505C cells	[226]
Lemon fruit extract	MCF-7 cells	[227]
Lemon seed extracts	MCF-7 cells	[228]
*Citrus bergamia* juice	SH-SY5Y cells	[229]
*Citrus bergamia* juice	HepG2 cells	[230]
*Citrus bergamia* juice	SK-*N*-SH/LAN-1 xenograft mice	[231]
Flavonoid-rich extract of bergamot juice	HT-29 cells	[232]
*Citrus reticulata* pericarpium extract	WEHI 3B cells	[244]
*Citrus reticulata* Blanco peel extract	SNU-668 cells	[245]
*Citrus aurantium* peel extract	A549 cells	[246]
*Citrus aurantium* peel extract	U937 cells	[247]
Orange peel extract	C57Bl/6 mice	[55]
Orange peel extract	Apc^(Min/+)^ mice	[54]
*Citrus aurantifolia* peel extract	MCF-7 cells	[248]
*Citrus paradis* peel extract	Kasumi-1 cells	[249]
*Citrus bergamia* essential oil	SH-SY5Y cells	[250]
*Citrus bergamia* essential oil	SH-SY5Y cells	[251]

AOM: azoxymethane; DMBA: 7,12-dimethylbenz(α)anthracene; NNK: 4-(methyl-nitrosoamino)-1-(3-pyridyl)-1-butanone.

**Table 6:** The main epidemiological and clinical studies, systematic review, and meta-analysis on the anti-cancer effects of *Citrus* fruits.

Study Design	Subjects	Reference
Case–control study	935 nasopharyngeal carcinoma (NPC) patients aged 15 to 74 years and 1032 community controls	[252]
Case–control study	304 esophagus squamous cell carcinoma patients and 743 hospital controls	[253]
Cohort study	120,852 Dutch men and women aged 55–69	[254]
Case–control study	512 men and 86 women with cancer of the oral cavity and pharynx and 1008 men and 483 women controls	[255]
Case–control study	527 incident, histologically confirmed cases and 1297 frequency-matched controls	[256]
Prospective study	42,311 US men	[257]
Case–control study	217 people with gastric cancer and 394 controls	[259]
Population-based case–control study	1459 incident breast cancer cases and 1556 frequency-matched controls	[264]
Clinic-based case–control study	384 cases of pancreatic cancer and 983 controls	[266]
Population-based case–control study	532 cases of pancreatic cancer and 1701 controls	[267]
Case–control study	130 incident patients with adenocarcinoma of the prostate and 274 controls	[268]
Hospital-based case–control study	304 incident cases of cutaneous melanoma and 305 controls	[269]
Cohort Study	42,470 Japanese adults with age ranging fron 40 to 79 years	[270]
Population-based case–control study	876 male patients with laryngeal/hypopharyngeal carcinoma	[271]
Systematic review	Stomach cancer	[272]
Systematic review	Pancreatic cancer	[273]
Systematic review	Breast cancer	[274]
Meta-analysis	Bladder cancer	[275]
Systematic review and meta-analysis	Bladder cancer	[276]
Meta-analysis	Bladder cancer	[277]
Meta-analysis	Esophageal cancer	[278]
Systematic review	Esophageal and gastric cancers	[279]
